# Research Progress in the Field of Gambogic Acid and Its Derivatives as Antineoplastic Drugs

**DOI:** 10.3390/molecules27092937

**Published:** 2022-05-04

**Authors:** Meng Li, Fali Su, Mingtao Zhu, Huan Zhang, Yuxin Wei, Yang Zhao, Jianmin Li, Shaowa Lv

**Affiliations:** 1 Heilongjiang University of Chinese Medicine, Harbin 150040, China; sx474401317@163.com; 2 Key Laboratory of Chinese Materia Medica (Ministry of Education), Heilongjiang University of Chinese Medicine, Harbin 150040, China; sfl20220330@163.com (F.S.); zmingt180@163.com (M.Z.); zgcdgxzh@163.com (H.Z.); wyx1017080239@163.com (Y.W.); 18745077225@163.com (Y.Z.); 3 The First Affiliated Hospital of Heilongjiang University of Chinese Medicine, Harbin 150040, China; fbhkfbhk@163.com

**Keywords:** Gambogic acid, structural modification, derivative, antitumor activity, polymer prodrug

## Abstract

Gambogic acid (GA) is a natural product with a wide range of pharmacological properties. It plays an important role in inhibiting tumor growth. A large number of GA derivatives have been designed and prepared to improve its shortcomings, such as poor water solubility, low bioavailability, poor stability, and adverse drug effects. So far, GA has been utilized to develop a variety of active derivatives with improved water solubility and bioavailability through structural modification. This article summarized the progress in pharmaceutical chemistry of GA derivatives to provide a reference and basis for further study on structural modifications of GA and expansion of its clinical applications.

## 1. Introduction

**Figure 1:**
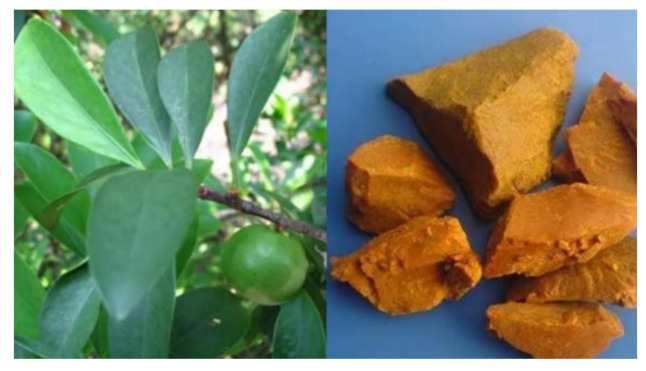
Garcinia plant and its dried resin.

**Figure 2:**
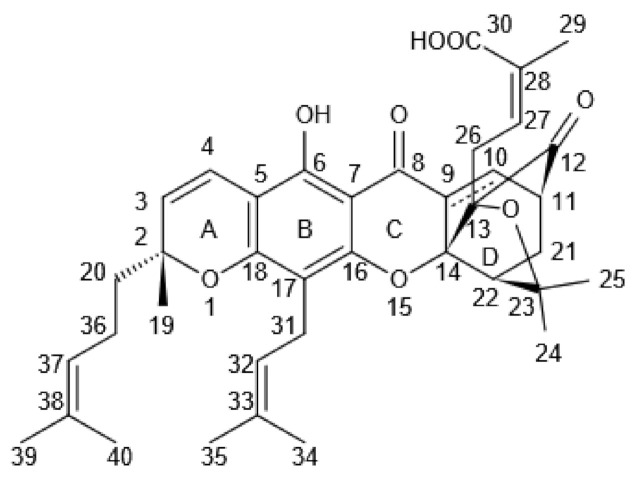
Structure of Gambogic acid (GA).

The introduction of synthetic modifications in the active structure of natural compounds is an effective means of generating clinically effective therapeutic drugs. Hence, the derivatization of natural pharmaceuticals has been successfully implemented in the clinical field [1]. Gambogic acid (GA) (Figure 1) is traditionally used as a herbal medicine, mainly for its anti-inflammatory and hemostatic properties. GA-producing Garcinia plants can be frequently spotted in Southern China, Vietnam, Cambodia, and Thailand [2]. The gamboge resin is used as a coloring material, as well as a therapeutic drug in traditional Chinese medicine (TCM) to treat human diseases [3]. GA (Figure 2) is an organic acid extracted from the dried resin of the *Garcinia mandshurica* plant. It belongs to cymbal ketones containing xanthone nuclei and a unique 4-oxa tricyclic [4.3.1.0^3,7^] decane-2-one scaffold [4]. This structure has the characteristics of easy ring opening, poor water solubility, alkali resistance, and poor thermal stability. Studies have shown that GA has several pharmacological effects such as anticancer, antiviral, anti-inflammatory, and anti-infective [5,6]. Presently, the research on GA focuses on its antitumorigenic property and has been characterized to be used in the treatment of a wide range of cancers, including lung, liver, melanoma, prostate, cervical, ovarian, and kidney cancers [7,8,9,10,11,12,13].

The action mechanism of GA is complex, which may be due to its complex molecular structure that causes it to have multiple molecular targets. Some studies have shown that GA upregulates pro-apoptotic genes (Bax, p53, DIO-1, SRC-3) and downregulates apoptosis inhibitory genes (Bcl-2, NF-κB, surviving, TfR), and it can cause cell cycle arrest, telomerase inhibition, downregulation of the AKT signal pathway, and the inhibition of AKT phosphorylation. Activation of apoptosis-related protein caspase-8/3, alteration of PI3K/AKT/P21 signal transduction, transferrin binding, and NF-κB regulation, and other mechanisms to treat cancer diseases are investigated in [14,15,16,17,18]. In addition, some studies have shown that GA can regulate the ratio of angiotensin converting enzyme 1/angiotensin, converting enzyme 2, and inhibit the proliferation effect of transforming growth factor β1 and CoCl_2_ on HLF1, and reduce its expression [19].

However, certain application-oriented shortcomings of GA, such as poor solubility, toxicity, short half-life, and poor stability, limit its clinical applications [20]. Reasonable drug modifications of GA can improve its physical and chemical properties, leading to increased drug potency. GA structural modifications occur in the hydroxyl group at C-6; in the C-9/10, C-32/33, C-37/38 double bonds; in the C-30 carboxyl group; and in the C34/39 allyl group [21]. Several GA derivatives have shown a better antitumorigenic effect than GA itself.

In addition, polymer prodrug nanomicelles containing hydrophobic drugs have attracted more and more attention. Hydrophobic drugs and hydrophilic polymer molecules with many advantages, such as degradability, biocompatibility, targeting, and tissue permeability, are combined by covalent bonds and self-assembled to form polymer prodrug nanomicelles. The new dosage forms can improve the solubility of hydrophobic drugs and selectively deliver drugs through a variety of response strategies [22,23,24,25]. In this paper, articles in related fields were found through the PUBMED, CNKI, and Sciencedirect databases, and the past advances in pharmacochemistry of GA derivatives were briefly summarized in order to provide a reference for improving the activity of the preclinical development of antineoplastic drugs.

## 2. Structural Modifications of GA

GA belongs to caged xanthone compounds, which contain a number of active chemical modification sites, for example, the carboxyl group at the C-30 position can undergo corresponding conversion reactions to yield new GA derivatives.

### 2.1. Addition Reaction of C9-C10 Double Bond

**Figure 3:**
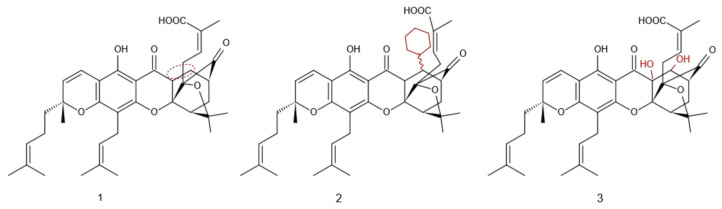
Structure of Gambogic acid derivatives at C9-C10.

Zhang et al. [26] found that the pharmacological activity of a modified product, obtained by either hydrogenating or cyclizing the C9-C10 double bond, was much weaker than that of GA itself. The authors used L-selectride for selective reduction of the 9/10 double bond in GA to obtain compound **1** (Figure 3). GA reacted with the cyclohexyl cuprate prepared from cyclohexyl magnesium chloride and iodocopper (CuI) to form compound **2** (Figure 3), which showed antitumor activity that was more than 10 times lower than that of GA. The inhibitory effects of compound **3** (Figure 3) on A549, BGC823, SKOV3, HT-29, and Bel7402 cancer cell lines were much weaker than those of GA [27]. The introduction of the polar group at the C9-C10 double bond increased the solubility of GA but decreased its antitumor activity, indicating that the 10th-position double bond in GA could be an essential structural moiety for its antitumor activity.

Based on the above studies, Seo et al. [28] hypothesized that the 9,10 double bond in the α,β-unsaturated ketone of GA might act as an electrophilic Michael addition center to covalently modify the nucleophilic cysteine mercaptan of several target proteins. The experimental results showed that the covalent modification of intracellular protein free mercaptan groups induced by GA interfered with the formation of proper disulfide bonds in the process of protein folding. It also induced the accumulation of misfolded proteins in the endoplasmic reticulum and mitochondria, resulting in the pressure and expansion of these organelles, resulting in cell death associated with vacuolation of cancer cells.

### 2.2. Structural Modifications of the Carbon–Carbon Double Bonds at C-32/33 and C-37/38

**Figure 4:**
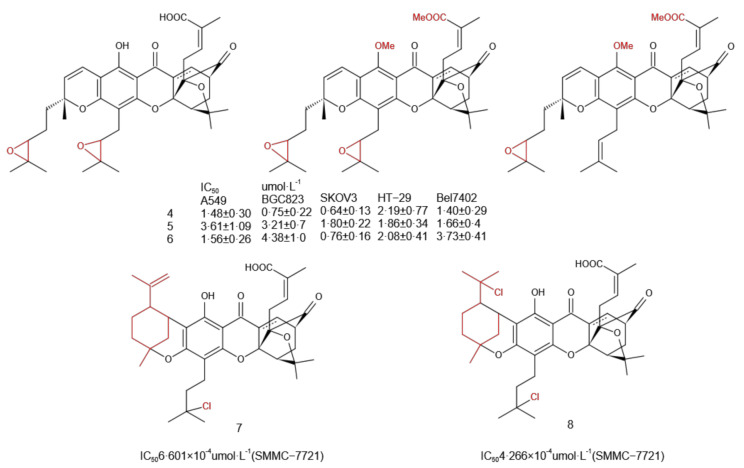
Structural modifications of the carbon–carbon double bonds at C-32/33 and C-37/38.

Wang et al. [27] synthesized a series of promising antitumor drugs (Figure 4) by converting the carbon–carbon double bonds at the C-32/33 and C-37/38 positions into epoxy groups. Compounds **4**, **5**, and **6** showed significantly stronger cytotoxic effects than that of GA in A549, BGC823, SKOV3, HT-29, and Bel7402 cells. Compounds **7** and **8** (Figure 4), synthesized by Feng et al. [29], showed stronger inhibitory effects on the growth of human hepatocellular carcinoma (SMMC-7721) cells than that of GA. The initial screening of synthetic modifications revealed that the introduction of two chlorine atoms could have potent antitumor effects, possibly due to the electrostatic interaction between the negatively charged chloride group and positively charged tumor cells.

### 2.3. Structural Modification at the C-34/39 Allylic Position

**Figure 5:**
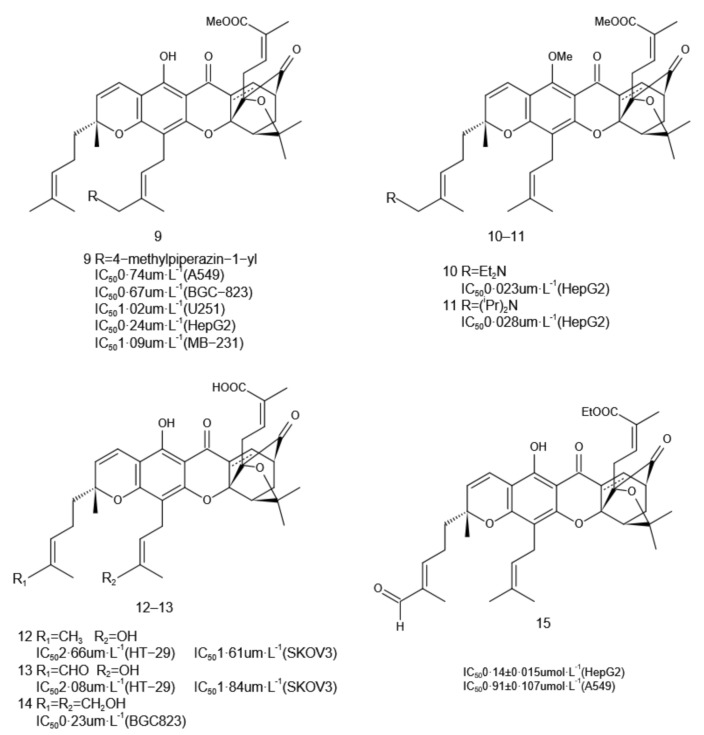
Structural modifications at the C-34/39 allylic position.

The most common modification site of GA is the C-34/39 allylic group. The antitumor activity of the compound can be significantly improved by introducing specific modifications at C-34, C-39, or both of these locations in GA. Zhang et al. [21] reported a series of GA derivates produced by introducing hydrophilic aliphatic and aromatic amines into the C-34 allyl site of GA. Compound **9** (Figure 5) with a hydrophilic 4-methylpiperazine-1 group exhibited the best effect on A549, BGC-823, U251, HepG2, and MB-231 cells with IC_50_ values of 0.74, 0.67, 1.02, 0.24, and 1.09 µmL^−1^, respectively. Thus, the introduction of a hydrophilic aliphatic amino group at C-34 in GA could increase its water solubility and antitumor activity. To find reasonable chemical modification sites and effective groups, Sun et al. [4] introduced aliphatic amine, aromatic amino, alkoxy, and halogen at position C-39 in GA to derive a series of compounds. The selectivity of aliphatic amino compounds **10** and **11** (Figure 5) to HepG2 was greatly improved, and the corresponding IC_50_ values were 0.023 and 0.028 µmL^−1^, respectively, which were almost 10 times higher than that of GA.

Wang et al. [27] introduced hydroxyl at positions C-34/39, and synthesized compounds **12**–**14** (Figure 5). The cytotoxicity test in SKOV3 cells showed both compounds **12** and **13** were 2–3 times more cytotoxic than that of GA. The toxic activity of compound **14** in the BGC-823 cell line was almost 20 times higher than that of GA, but its inhibitory effects on other tumor cells were equal to or weaker than that of GA. Thus, it was suggested that the inhibitory effect of the same group on different tumor cells could be significantly different.

On the basis of a large number of reports on the structural modification of C-34 and C-39 in GA, Guo [30] et al. modified the isoprene side chain of the A ring on GA to obtain compound **15**. The results showed that compound **15** had a stronger cytotoxic effect on A549 and HepG2 cancer cells than GA, and inducing apoptosis in HepG2 cells might be related to the expression of Bcl2 and Bax.

### 2.4. C-30 Structure Modification

**Figure 6:**
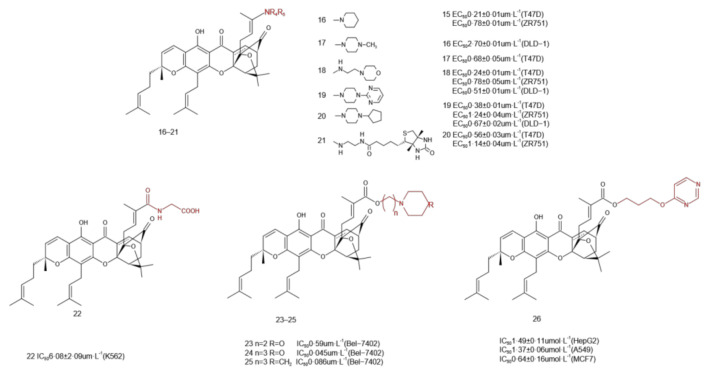
C-30 structure modification.

Zhang et al. [26] coupled various amines in the presence of 4-dimethylaminopyridine (DMAP) and 1-ethyl-3-(3-dimethylaminopropyl)carbodiimide (EDC) to form the corresponding amides **16**–**21** (Figure 6). Three tumor cell lines, T47D, ZR751, and DLD-1, were used to estimate the cytotoxic effects of GA derivatives by the HTS caspase activation test. The results showed that compound **16** had the strongest activity against T47D and ZR751 cells, while compound **17** was the most effective against DLD-1 cells. Through C-30 amidation of GA [31], we could increase the water solubility of compound **22** (Figure 6). The cytotoxicity test further revealed that compound **22** had toxic effects on several other types of tumor cells and was even effective against multidrug-resistant cancer cells. Compound **22** induced apoptosis by activating caspase-3/8/9 via Bax upregulation and Bcl-2 downregulation. To improve the water solubility of GA, He et al. [32] introduced different kinds of amide alcohols at the C-30 position. Compounds **22**, **23**, and **24** (Figure 6) were the most potential candidates for inducing cytotoxicity in Bel-7402 cells (the IC_50_ values were 0.59, 0.045, and 0.086 μmol·L^−1^, respectively). It is worth noting that the alkyl alcohol length of the connecting amine and type of cyclic amine (such as morpholine, piperidine, piperazine, and N-hydroxyethyl piperazine) was very important for their anticancer activities in vitro, and the compounds with trialkanol linker had stronger anticancer activity.

GA inhibits the activity of IκB Kinase-beta (IKKβ) by inhibiting the activation of the TNFα/NF-κB pathway, which leads to the apoptosis of cancer cells [33]. As a polar group, pyrimidine is a very important weakly basic nitrogen-containing aromatic heterocyclic compound, which plays a physiological role by forming hydrogen bonds with biological receptors [34]. Based on the above thinking, Ling et al. [35] linked alkyl connectors with pyrimidine heterocycles and modified the carboxyl group at position 30 in GA to obtain a new GA derivative **26**. The experimental results showed that its anticancer activity and water solubility were greatly improved. The introduction of pyrimidine could also improve the binding affinity between GA and IKKβ and induce cell apoptosis more effectively. Compound **26** might be an efficient and selective candidate for anticancer drugs.

### 2.5. Diversification of Modification Sites

Due to the complex structure of GA with several active modification sites, compounds derived after synthetic modification at a single site could not maximize their water solubility and pharmacological activities. Therefore, the simultaneous introduction of multiple active groups could significantly help overcome the shortcomings of GA’s poor water solubility, stability, and efficacy.

**Figure 7:**
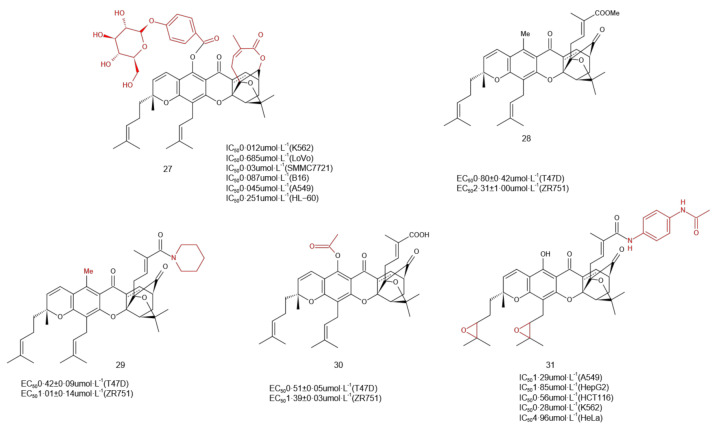
Diversity of modification sites.

One of the ways to improve the physicochemical properties of flavonoids is to combine them with sugar molecules to form the corresponding glycosides [36]. Glycosylation affects the solubility, stability, and biological activity of aglycones. Zhou et al. [37] applied synthetic strategies to reduce the C-12 carbonyl group, form a ring with the C-30 carboxyl group, and introduce a more polar glycoside group at the C-6 position in GA to yield the target product **27** (Figure 7). The results of an in vitro antitumor activity assay showed the cytotoxicity of Rattan glycoside **27** against six tumor cell lines, K562, SMMC7721, A549, LoVo, HL-60, and B16, was stronger than that of GA, and the corresponding inhibitory activities are shown in Figure 7. The overall molecular polarity and activity of the compound were significantly enhanced, and its IC_50_ was 10–50 times higher than that of GA. Therefore, based on the idea of retaining the GA bridged ring structure, the activity of derivates could be significantly increased by enhancing their polarities.

Compounds **28**, **29**, and **30** were obtained by methylation or acylation of 6-OH [26], and their antitumor activities against T47D, ZR751, and DLD-1 cell lines were quite similar to that of GA. Hence, the apoptosis-inducing activity of 6-hydroxyl group modification on GA was not important.

Chen [38] et al. obtained a series of compounds by modifying the C-30 carboxyl group and carbon–carbon double bonds at C-32/33 and C-37/38 in GA, and evaluated the anti-angiogenic properties of the compounds through a zebrafish model of activity and toxicity, the experimental results showed that compound **31,** obtained by acylation of the carboxyl group at C-30 and epoxidation of the carbon–carbon double bonds at C-32/33 and C-37/38, could effectively inhibit neogenesis in a concentration-dependent manner with vascular formation and less toxicity than GA, suggesting that it might serve as a potential novel low-toxicity angiogenesis inhibitor.

### 2.6. Structural Modification of the A Ring

**Figure 8:**
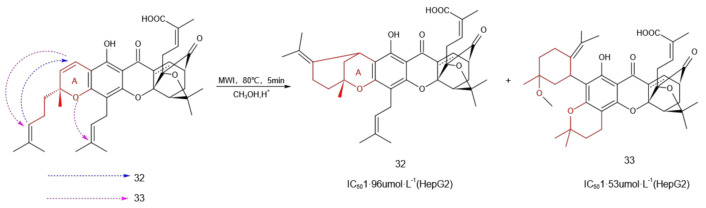
Structural modification of the A ring.

Since the A ring in GA does not have a direct modification site, there are only a few reports on A-ring modification. Therefore, it was very important to explore the derivatives of the A ring to study their antitumor properties. Under microwave irradiation (200 W, 80 °C, 5 min) [39] and using a combination of small amounts of sulfuric acid (H_2_SO_4_) and methanol as a solvent, the intramolecular cyclization reaction was performed on GA to obtain compounds **32** and **33** (Figure 8). These showed tumor growth inhibition through the activation of the apoptosis pathway, and both had stronger antiproliferative activities against HepG2 cells than that of GA.

### 2.7. The Simplified Structure of GA

For some natural products with complex chemical structures, it is possible to identify simpler but effective groups by intercepting parts of the structure. It is also easier to produce small-molecule substances with promising anticancer properties by chemical synthesis. Studies have shown that by simplifying the structure of GA and then introducing pharmacophores, the antitumor activity, as well as the oral bioavailability of the drug, can be improved.

**Figure 9:**
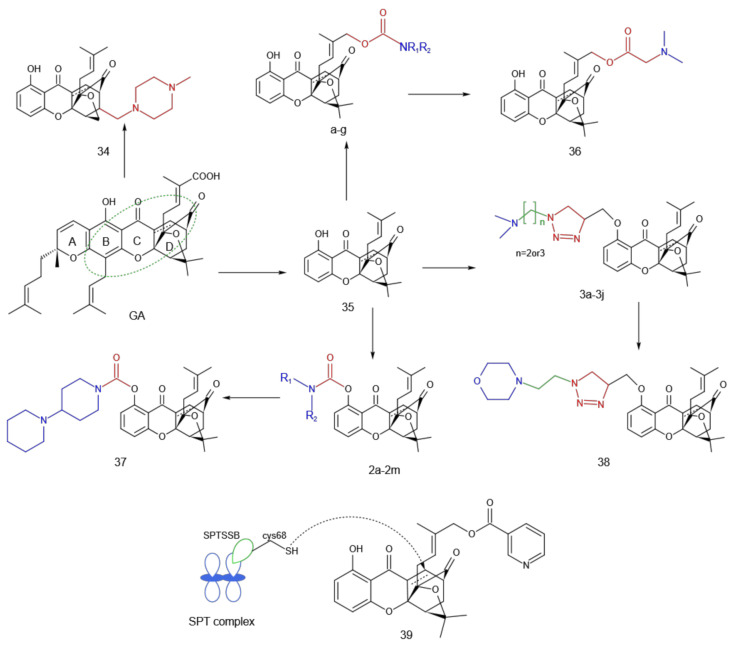
Simplified structure of GA.

The α,β-unsaturated ketone moiety of the CD ring cage and its BC plane region are required for the antitumor activity of GA, and the peripheral portion is suitable as a site for various modifications [26,40,41]. Zhang et al. [42] synthesized xanthone compound **34** based on the regioselective propargylation of different hydroxyflavonoid substrates (Figure 9). The cytotoxicity test showed that the length and bulkiness of the substituted moiety in the cage region significantly affected their pharmacological activities. The in vivo antitumor activity test via oral administration estimated that the tumor growth inhibition rate of compound **34** was 57.74%, which was a novel oral antitumor drug. Compound **35** had good antitumor activity in vitro, but a poor in vivo effect. Based on **35**, Wu et al. [43] synthetically introduced a hydrophilic nitrogen-containing heterocycle into the isopentenyl group of the D ring. A series of derivatives a–g (Figure 9) were obtained, amongst which compound **36** had a stronger inhibitory effect on the growth of Heps, inoculated in mice in vivo, and the in vitro cytotoxicity in HepG2 cells was similar to that of compound **35**. The MTT assay showed that the compounds obtained by linking the nitrogen-containing heterocycle to the ester group through a methylene group significantly affected the cytotoxicity of cancer cells compared with the direct linking of the nitrogen-containing heterocycle to the ester group. Therefore, appropriate changes in the length of the carbon chain and the size of the nitrogen-containing heterocycle might significantly affect the cytotoxicity in subsequent studies.

Xu et al. [44] simplified the GA structure, retained the BCD ring, and modified the hydroxyl group of the B ring to obtain a series of carbamate caged anthrones 2a–2m. MTT analysis showed that these compounds had considerable antiproliferative activities and better physicochemical properties. Dipiperidine carbamate was introduced into the hydroxyl group of the B ring to obtain compound **37** (Figure 9). By inhibiting the Hsp90 ATPase, Hsp90 protein was degraded, thereby inhibiting tumor cell proliferation and angiogenesis.

The copper-catalyzed 1-3-dipolar cycloaddition reaction-mediated coupling of alkynes and azides to form 1,2,3-triazole rings commonly referred to as “click chemistry” due to its ease of use, has become a fast and efficient method for the construction of pharmacologically active compounds [45,46]. The 1,2,3-triazole ring is chemically stable against both acidic and basic hydrolysis and relatively resistant to metabolic degradation. Li et al. [47] combined the hydrophilic group and the caged anthrone-pharmacophore to synthesize a series of new triazole-containing caged anthrones 3a–3g. Compound **38** (Figure 9) exhibited significant inhibitory activities in vitro with IC_50_ values of 0.31 ± 0.02, 0.42 ± 0.05, 0.33 ± 0.07, and 0.28 ± 0.03 μmol·L^−1^, respectively, against the lung cancer cell model A549, A549/paclitaxel, A549/cisplatin, and HCT116 cells. It is important to note that the oral administration of compound **38** exhibited a growth inhibition rate of 66.43% in A549 transplanted mice. From these observations, it could be inferred that the combination of acid-resistant and alkali-resistant hydrophilic groups and pharmacophore groups could effectively reduce the first-pass effect of the gastrointestinal tract and show a significant antitumor effect.

Hoch et al. [48] showed that GA can bind to serine palmitoyltransferase complex (SPTSSB), thereby reducing sphingolipid levels in mice, thereby slowing or interrupting cancer-promoting overgrowth. Their team synthesized xanthone compound **39** by the synthetic method of Wang, Wu et al. [43,49]. The experimental results showed that compound **39** could also bind to SPTSSB with more selectivity, reduce the phosphorylation of the oncogenic kinase AKT, and decrease de novo synthesis of the oncogenes transforming growth factor b (TGF-b), c-Fos and jun-B. To prove the electrophilic moiety bound to SPTSSB, the C=C bond in compound **18** was reduced, and it was found that the resulting new compound could not competitively bind to SPTSSB, proving that the electrophilic moiety was an a,b-unsaturated ketone.

### 2.8. Generation of Polymer Prodrugs

**Figure 10:**
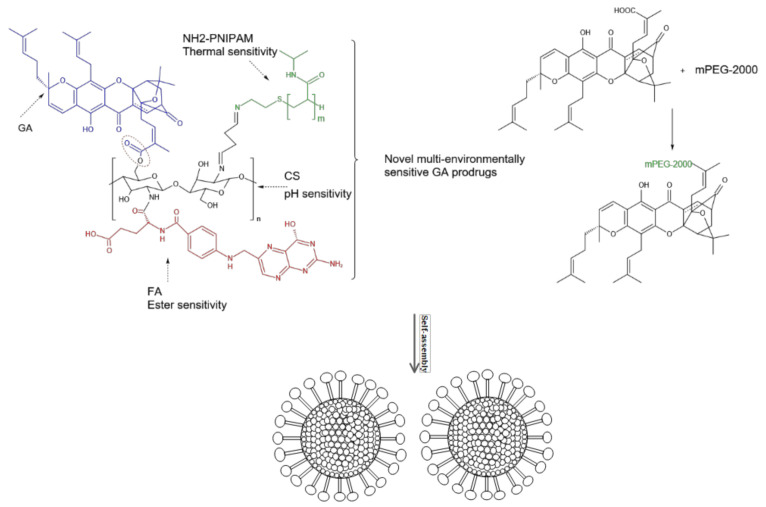
GA prodrug nano micelles.

Covalently bonded hydrophilic polymers and self-assembled hydrophobic drug-polymer prodrug micelles (PPMs) have been shown to increase the selectivity and solubility of hydrophobic drugs, thus solving the problem of low drug loading [23,24,25]. Du et al. [50] prepared a folic acid-chitosan (FA-CS) complex with amino-terminated heat-sensitive poly (N-isopropylacrylamide) (NH_2_-PNIPAM) to form a pH and temperature-sensitive FA-CSPN system. The multi-environment sensitive prodrug nanomicelle GFCP (Figure 10) was synthesized by esterification of GA with FA-CSPN. In vivo antitumor activity measurements showed that compared with GA alone, this combination therapy could significantly inhibit tumor growth and reduce side effects in vivo. Thus, the amphiphilic compound formed by esterification between hydrophilic chitosan conjugate and lipophilic GA could achieve not only high drug loading and controlled release but also fixed-time drug release in the tumor. Cai et al. [51] coupled mPEG-2000 to GA by esterification and formed amphiphilic polymeric nanomicelles (Figure 10) by self-assembly. In vivo antitumor studies have shown that GA-mPEG2000 micelles are more effective in inhibiting tumor growth and prolonging the lives of mice with tumors, which may be because GA-mPEG2000 polymer micelles protect ester bonds from hydrolysis, and nanomicelles can penetrate the leaking vascular system and specifically accumulate in tumor tissues through enhanced permeability and retention effects, resulting in the slow release of drugs at tumor sites. This would in turn, increase the local drug concentration within tumor cells compared to other non-cancer tissues.

## 3. Conclusions and Prospects

Based on the above-mentioned findings, the α, β-unsaturated ketone moiety of the CD ring cage region and its BC plane region were necessary for the antitumor activity of GA. The structural modification of GA mainly occurs at the C-30 carboxyl group and the C-34/39 allyl position. The introduction of aliphatic amines and hydrophilic alkanolamines into the carboxyl group at position C-30 could significantly increase the water solubility of the respective compound. The alkyl alcohol length of the connecting amine part and the type of cyclic amines were very important for its anticancer activity. Trialkanols and morpholine ring compounds showed stronger anticancer activities. Combined with a hydrophilic polymer, this site could be self-assembled to form prodrug nanomicelles, which could not only increase the solubility but also achieve sustained release and targeting specificity of the drug, which is a very promising idea for structural modification. The introduction of hydrophilic aliphatic amines at the allylic position of C-34/39 was beneficial to improving the antitumor activity and solubility of GA. The activity of the compound obtained after the modification of the hydroxyl group at the C-6 position was comparable to that of GA, but the multi-position modifications including the hydroxyl group at the C-6 position could yield a compound with better water solubility and antitumor activity. Introducing glycoside fragments at modifiable sites might also be a feasible method to increase the water solubility of the compound. Additionally, given the requirements of increasing solubility and enhancing activity, introducing different groups at different GA modification sites at the same time could also be an effective method for highly promising antitumor drugs.

Active natural products have unique structural diversities and complexities that play an important role in their clinical applications for the treatment of various diseases. In the clinic, most antineoplastic drugs are modified natural products or natural products by themselves. Most antineoplastic drugs have the problems of high toxicity and low solubility. Structural modification is one of the effective measures to solve these problems. As an active natural product, GA has great potential in cancer treatment. New compounds with higher bioavailabilities and antitumor activities can be obtained by structural modifications of the original natural compound. However, due to a series of complex synthetic reactions, several by-products are most likely to form, thus causing the yield of the target product due to the loss of compounds at each separation and purification step. New drug research and development needs clinical trials to prove the safety and effectiveness of these synthetically derived compounds, and the high cost of drug development research is also a problem in obtaining structurally modified novel drugs.

The structure of GA is complex and may limit its absorption in the intestinal tract to enter the blood circulation. Therefore, prodrug strategies and special drug delivery systems may provide unique methods for the clinical application of GA and its derivatives. It is ideal to develop nanopharmaceuticals through structural modifications to generate final drug candidates. It is extremely difficult to synthesize new drugs using synthetic chemistry alone. With the deepening of research and rapid development of science and technology, new technologies conducive to large-scale production, such as biosynthetic modification, will continue to be applied to the synthesis of GA and its derivatives, thus providing promising opportunities for designing new structures and optimizing lead compounds derived from natural products. This strategy will be highly beneficial in developing novel antineoplastic drugs and produce good social and economic benefits.
